# An affordable method to obtain cultured endothelial cells from peripheral blood

**DOI:** 10.1111/jcmm.12133

**Published:** 2013-10-01

**Authors:** Carlos Bueno-Betí, Susana Novella, Macarena Lázaro-Franco, Daniel Pérez-Cremades, Magda Heras, Juan Sanchís, Carlos Hermenegildo

**Affiliations:** a Research Foundation, Hospital Clínico of Valencia – INCLIVAValencia, Spain; b Department of Physiology, University of ValenciaValencia, Spain; c Institut d'Investigacions Biomèdiques August Pi i Sunyer (IDIBAPS) Institut Clinic de Tòrax, Hospital ClinicBarcelona, Spain; d Cardiology Department Hospital Clínico of Valencia and Medicine Department, Universitat de ValènciaValencia, Spain

**Keywords:** endothelial progenitor cells, cell culture, vasculogenesis

## Abstract

The culture of endothelial progenitor cells (EPC) provides an excellent tool to research on EPC biology and vascular regeneration and vasculogenesis. The use of different protocols to obtain EPC cultures makes it difficult to obtain comparable results in different groups. This work offers a systematic comparison of the main variables of most commonly used protocols for EPC isolation, culture and functional evaluation. Peripheral blood samples from healthy individuals were recovered and mononuclear cells were cultured. Different recovery and culture conditions were tested: blood volume, blood anticoagulant, coating matrix and percentage of foetal bovine serum (FBS) in culture media. The success of culture procedure, first colonies of endothelial cells appearance time, correlation with number of circulating EPC (cEPC) and functional comparison with human umbilical vein endothelial cells (HUVEC) were studied. The use of heparin, a minimum blood volume of 30 ml, fibronectin as a coating matrix and endothelial growing media-2 supplemented with 20% FBS increased the success of obtaining EPC cultures up to 80% of the processed samples while reducing EPC colony appearance mean time to a minimum of 13 days. Blood samples exhibiting higher cEPC numbers resulted in reduced EPC colony appearance mean time. Cells isolated by using this combination were endothelial cell-like EPCs morphological and phenotypically. Functionally, cultured EPC showed decreased growing and vasculogenic capacity when compared to HUVEC. Thus, above-mentioned conditions allow the isolation and culture of EPC with smaller blood volumes and shorter times than currently used protocols.

## Introduction

Vasculogenesis in adults was for first time reported after the identification and characterization of a population of EPC derived from peripheral blood 1. Endothelial progenitor cells are incorporated into new vessels undergoing active angiogenesis. This process of post-natal vasculogenesis refers to the formation of new blood vessels from progenitor cells in the adult and has been associated with different physiological functions 2 and pathological disorders as cancer 3, cardiovascular diseases (CVD) 4–5 and diabetes 6.

Since EPC were discovered, a great deal of literature has emphasized the importance of such progenitor cells in the maintenance of endothelial integrity by both exerting a paracrine effect to promote angiogenesis and integrating themselves into new vessels 6–7. Actually, an inverse correlation between EPC numbers and cardiovascular risk factors has been reported in patients with cardiovascular risk, but no history of CVD 8.

Besides the number of EPCs, the progenitor cell function could be affected by individual factors, such as cardiovascular risk factors, different drug treatments, hormone levels and others 9. The study of EPC function requires obtaining EPC cultures, but one of the drawbacks existing is the lack of a simple and reproducible method to isolate, cultivate and expand EPC. It could be as a result of the small fraction at which these cells are found circulating level, representing only about 0.0001% of total mononuclear cells (MNC) of peripheral blood in human adults 10. Culture of EPC has been performed in several ways, for early and late EPC 11, although a systematic and detailed protocol to obtain replicate data on cultured EPC is still required.

Moreover, a potential clinical use of cultured EPC has been proposed, including re-endothelialization of injured vessels, reducing atherosclerotic disease appearance and progression and revascularization of infarcted regions 12. Endothelial progenitor cells have also been proposed as potential therapeutic tools for gene therapy in growing cancers 13.

In the present study, we evaluated the influence of different culture conditions in obtaining EPC cultures, and we tested EPC functional capacity and compared it with HUVEC, widely used as a laboratory model system for studies on endothelial cell function and pathology. Therefore, we proposed an affordable and reproducible method to obtain EPC cultures to perform functional cell assays.

## Materials and methods

### Study design

This study was designed to compare different culture conditions for the best way to isolate and to obtain cultured, functional EPC. First, we selected the optimal blood sample withdrawal conditions in terms of volume and anticoagulant. Second, the culture conditions (extracellular matrix, culture media composition) were analysed. Finally, the functional capacity of the cultured EPC (measured in terms of cell growth and adhesion, cell proliferation and vasculogenesis) was the parameter to ensure the best culture conditions of the obtained cells, taking HUVEC as standard reference.

### Blood samples

Peripheral blood samples were obtained from 60 healthy individuals at the Cardiology Service, Hospital Clinico of Valencia, in accordance with institutional guidelines. The characteristics of the participants enrolled are presented in Table S1.

Different blood volumes (60 ml) were withdrawn by venipuncture and were collected in tubes containing different anticoagulants: ethylenediaminetetraacetic acid (EDTA), heparin and sodium citrate (Vacutainer, Becton Dickinson, San Agustin del Guadalix, Madrid, Spain). To avoid sample contamination with mature endothelial cells (EC), first 6 ml of collected blood was discarded.

Blood samples were processed within 2 hr after extraction. To assess the effect of time sampling on EPC culture yields, a set of samples were left on a blood roller mixer at room temperature and were processed 24 hrs after withdrawal.

This investigation conforms to the principles outlined in the Declaration of Helsinki, was approved by the Ethical Committee of Clinical Research of the INCLIVA, Hospital Clinico of Valencia, Spain, and written informed consent was obtained from all donors.

### Mononuclear cell isolation

Mononuclear cells from peripheral blood samples were isolated as described before 14. Briefly, non-diluted blood was layered over Lymphoprep (Axis-shield, Oslo, Norway) in a volume ratio 2:1, and centrifuged at 800 rcf for 30 min. at room temperature. Mononuclear cells from interphase were collected and washed twice with 6% foetal bovine serum (FBS) (Gibco, Life technologies, Alcobendas, Spain) in Dulbeco's PBS solution (Gibco).

### Endothelial progenitor cell culture

Mononuclear cells isolated from blood samples were collected in endothelial growing media (EGM)-2 complete medium with the following composition (Lonza, Lonza Ibérica, Barcelona, Spain): Endothelial cell basal medium-2 supplemented with EGM Single Quots containing hydrocortisone, 2% FBS, hFGF-B, VEGF, R3-IGF-1, ascorbic acid, heparin, FBS, hEGF, and GA-1000 (gentamicin, amphotericin-B). Mononuclear cells were seeded onto fibronectin-treated plates (2.5 μg/cm^2^; Becton Dickinson) at a final density of 1.5 × 10^6^ cells/cm^2^. Twenty-four hours after seeding, non-adherent cells were removed and attached cells were further cultured at 37°C and 5% of CO_2_. Culture media was changed every 2 days until first EPC colonies appeared or up to a maximum of 40 days.

To test different culture conditions, some of the above-mentioned parameters were modified. In this study, we tested two different FBS media concentrations, 2% and 20%, as well as two different coating matrixes, fibronectin 2.5 μg/cm^2^ and Gelatin 1% (Sigma-Aldrich, Tres Cantos, Madrid, Spain).

### Human umbilical vein endothelial cell culture

Human umbilical vein endothelial cell culture was isolated by collagenase treatment of human umbilical veins from newborns as described earlier 15. Briefly, umbilical veins were flushed with sterile PBS solution (Sigma-Aldrich) to wash the clotted blood out and then perfused with 1% collagenase solution and incubated at 37°C for 15 min. Endothelial cells were recovered by centrifugation and seeded onto gelatin-treated 25 cm^2^ flasks (BioLite, LabClinics, Barcelona) in specific endothelial media EGM-2.

### Quantification of circulating EPC in peripheral blood

Peripheral blood samples were recovered in heparin tubes and processed within 2 hrs after extraction. Circulating EPC (cEPC) were stained with anti-human VEGFR2/kinase insert domain receptor (KDR) conjugated with phycoerythrin (PE; R&D Systems, Madrid, Spain), fluorescein isothiocyanate (FITC)-conjugated anti-human CD34 (Becton Dickinson) and peridinin chlorophyll protein complex (PerCP)-conjugated anti-human CD45 (Becton Dickinson) or with the appropriate isotype controls. Red blood cells were lysed (BD FACS Lysing solution, Becton Dickinson) for 10 min. and stained cells were detected with a FC5000 cytometer (Beckman-Coulter, Madrid, Spain) and results were analysed with Infinicyt software (Cytognos S.A., Salamanca, Spain). Circulating EPC were identified as negative for the leucocyte marker CD45, positive for the prototypical stem cell marker CD34 and positive for the endothelial cell marker KDR (CD45^−^CD34^+^KDR^+^) 11.

### Endothelial progenitor cell characterization

#### Flow cytometry phenotypic characterization

Cultured EPC obtained under the most suitable conditions were also characterized by flow cytometry. The expression of endothelial antigens KDR and CD31 (also known as platelet-endothelial cell adhesion molecule, PECAM-1), progenitor antigen CD34 and leucocyte antigen CD45 was assessed. Briefly, harvested cells were stained with PE-conjugated anti-human KDR, FITC-conjugated anti-human CD31 or FITC-conjugated anti-human CD34 and PerCP-conjugated anti-human CD45, or with the appropriate isotype controls. CD45, CD34 and CD31 antibodies were purchased from Becton Dickinson and KDR antibody from R&D Systems. Stained cells were detected with a FC5000 cytometer (Beckman-Coulter) and results were analysed with Infinicyt software (Cytognos S.A.). Cultured EPC were considered as negative for the leucocyte marker CD45, positive for the stem-cell marker CD34 and positive for the endothelial cell markers KDR and CD31 (CD45^−^CD34^+^KDR^+^CD31^+^).

#### Immunofluorescence characterization

The ability of isolated EPC to uptake acetylated low density lipoprotein (Ac-LDL) and to bind Ulex-lectin, the classical way to define endothelial cells, was performed as described earlier 16–17. Briefly, EPC were incubated with 1,1′-dioctadecyl-3,3,3′,3′- tetramethylindo-carbocyanine–labelled Ac-LDL (Dil-acLDL, Life Technologies, Alcobendas, Spain), fixed with 4% paraformaldehyde and incubated with fluorescein isothiocyanate labelled Ulex europaeus agglutinin (FITC-UEA-1, Sigma-Aldrich). Cell nuclei were stained with 4′,6-diamidino-2-phenylindole (DAPI, Sigma-Aldrich). Stained samples were observed on a confocal spectral Leica SP2 microscope (Leica, Barcelona, Spain). Pictures were taken with a 40× objective and shown at ×1200 magnification.

Endothelial progenitor cells cultures were also tested for von Willebrand Factor (vWF) expression. In brief, cells were fixed with 4% paraformaldehyde solution and permeabilized for with PBS 0.25% Triton X-100 solution. Cells were incubated first with rabbit polyclonal vWF antibody (Abcam, Cambridge, United Kingdom) and then with a DyLight 488 conjugated anti-rabbit secondary antibody (Abcam). Counter staining was achieved by incubating cells with DAPI. Images were obtained with an inverted fluorescence microscope Nikon Eclispe Ti (100× magnification).

### Functional parameters

#### Cell growth curve

1.5 × 10^4^ cells were seeded in EGM-2 media on each well, culture media was changed daily and the counting process was repeated every day for 6 days. Briefly, cells were detached by using 0.05% trypsin solution, recovered by centrifugation and resuspended with 100 μl of Tripan blue solution (Sigma-Aldrich). Cells were counted on a Neubauer modified chamber and total number of cells per well were plotted to calculate the lag period (latent period of no growth), the log phase (when cells underwent exponential growth), the plateau (when growth rate dropped close to zero) and the saturation density (at which the cell population reached the plateau phase).

#### Cell proliferation

Cell proliferation was measured by flow cytometry with propidium iodide (PI) staining (Immunostep, Salamanca, Spain) to quantify the content of DNA and the distribution of a cell population along the different phases of the cell cycle 18. Briefly, cells were starved for 48 hrs and then stimulated for 18 hrs with EGM-2 complete media. After incubation, cells were detached with a 0.05% trypsin solution, fixed with 70% ethanol and stained with PI-RNase solution (Immunostep). Stained cells were analysed with FC5000 cytometer (Beckman-Coulter) and Infinicyt software (Cytognos). Results are shown as percentage of cells undergoing DNA synthesis plus mitosis with respect to total cell number analysed.

#### Cell adhesion

Adhesion assays were performed on fibronectin (Becton Dickinson) treated dishes (2.5 μg/cm^2^). In brief, 5 × 10^4^ cells were seeded on dishes with a 4 mm^2^ grid (Nunc, Madrid, Spain) and incubated at 37°C and 5% of CO_2_. After 30 min., non-adherent cells were removed and adhered cells were counted in six random squares by two independent observers. Data were expressed as a percentage of adhered cells relative to the total number of seeded cells.

#### Vasculogenesis assay

Vasculogenesis was analysed in Matrigel (Becton Dickinson) as previously described 19. In brief, Matrigel was diluted with EGM-2 media SBF free and allowed to solidify for 30 min. at 37°C. Thereafter, 1.5 × 10^5^ cells/well were seeded and incubated for 8 hrs. Then, pictures were taken with a Nikon Eclipse-Ti inverted microscope (Nikon, Izasa, Valencia, Spain) with 4× objective (total magnification 40×) and images recorded by Nikon digital sight Ds-QiMc camera.

Images from five different random fields per well were processed and analysed with Image Pro-Plus Software V.6 (Media Cybernetics, Rockville, MD, USA). Total length data of the tube-like structures were expressed in micrometers (μm).

### Statistical analysis

Values shown in the text and figures are mean ± SEM. For frequency comparison, contingency tables were analysed by Chi-squared test. Data normality was assessed by Kolmogorov–Smirnov test. Statistical comparisons were performed with one-way anova for multiple comparisons and then Bonferroni's test was performed. Student's *t*-test was applied for single comparisons. Correlation analysis was performed by linear regression. Growth curve data were analysed by two-way anova with Bonferroni's post-test. *P* < 0.05 was considered significant. The statistical analysis was carried out by using the Prism 5.04 software (GraphPad Software Inc., San Diego, CA, USA).

## Results

### EPC isolation conditions

Three clinically used anticoagulants were tested to study how the anticoagulant used for blood sampling influences EPC isolation and culture procedure. Identical blood volumes were recovered in tubes containing EDTA, sodium heparin and sodium citrate. Mononuclear cells from these samples were cultured and both the percentage of EPC cultures and the appearance mean time for the first EPC colonies were recorded.

**Figure 1:**
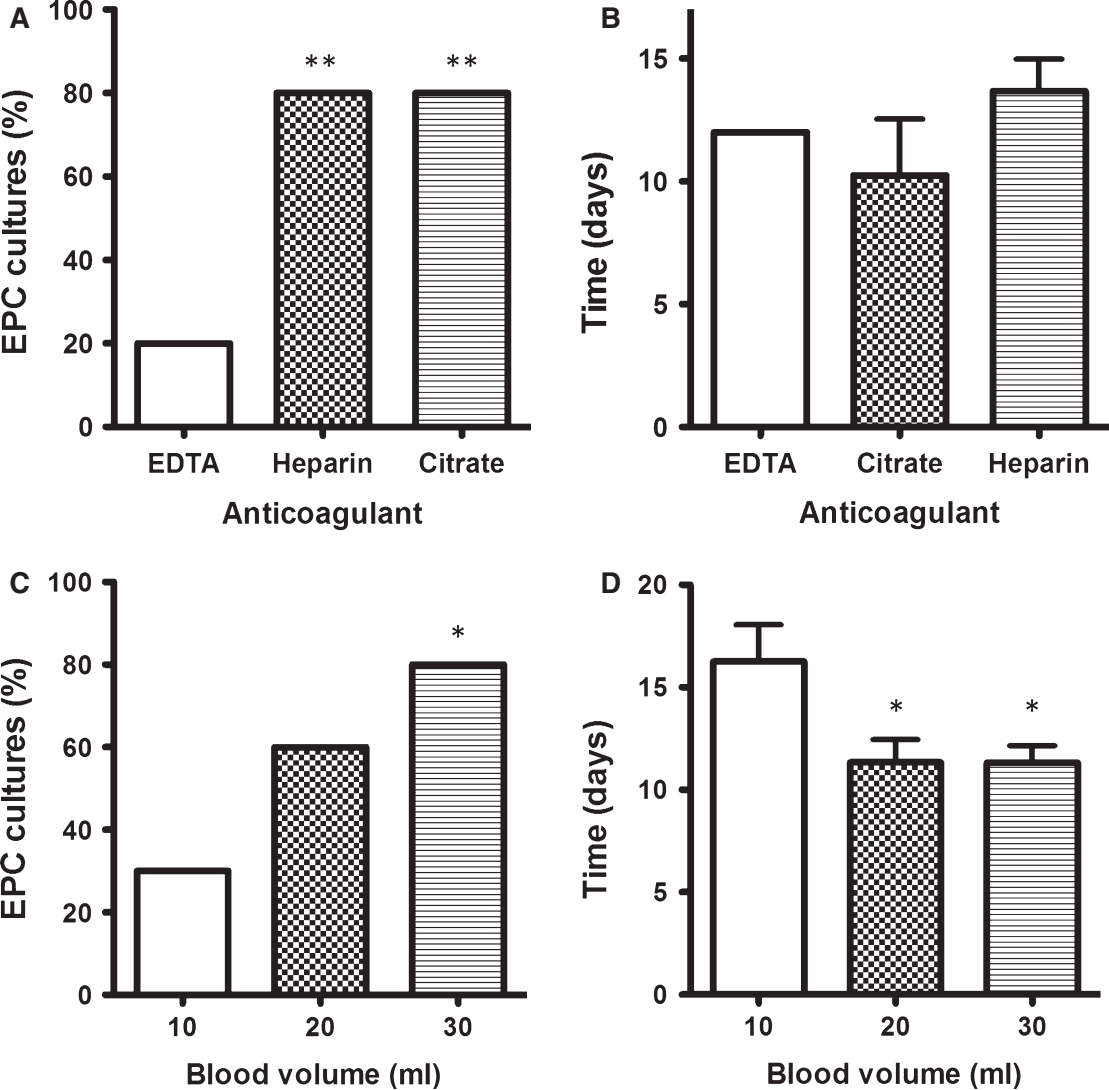
Endothelial progenitor cells' (EPC) isolation conditions. (A) Effect of anticoagulant in the success of EPC cultures and (B) the time at which it was possible to observe the first EPC colonies. Blood samples were collected in tubes containing three different anticoagulants: ethylenediaminetetraacetic acid (EDTA), heparin and sodium citrate. EPC initial cultures were followed up to 40 days of culture (**P < 0.01 *versus* EDTA by Chi-squared test; *n* = 10). Effect of blood volume in the success of EPC cultures: bars represent (C) the percentages of EPC cultures obtained from mononuclear cells obtained from different blood volumes and (D) the time at which it was possible to observe the first EPC colonies (*P *<* 0.05 *versus* 10 ml; *n* = 10).

Only 20% of EDTA-recovered blood samples gave rise to EPC cultures. However, when heparin or sodium citrate was used for blood recovery, 80% of EPC cultures were successfully obtained (*P* < 0.01; Fig. 1A). No significant differences were found with regard to EPC appearance mean time between anticoagulants (Fig. 1B). Moreover, 100% of cultures derived from citrated samples showed different morphology, were negative for the uptake of Ac-LDL and unable to bind Ulex-lectin, thus demonstrating they were not EPC (Fig. S1). Only heparin allows obtaining high yields of homogeneous EPC cultures with the shorter appearance mean time. Thus, rest of the experiments were performed with blood obtained with heparin as anticoagulant.

To assess the effect of blood volume on EPC isolation procedure, three different blood volumes (10, 20 and 30 ml) were tested. The highest EPC culture yields, referred as the percentage of EPC cultures obtained from blood samples, were found for blood samples of 30 ml (80%; *P* < 0.05 *versus* 10 ml). Although there were no statistically significant differences between samples of 20 and 30 ml, the 20% yield reduction in 20 ml blood samples is worth considering (Fig. 1B). The use of higher blood volumes for EPC isolation showed a decrease in EPC appearance mean time, from 16 days for 10 ml blood samples to 12 days for both 20 and 30 ml blood samples (*P* < 0.05; Fig. 1C). On the basis of obtained results, we chose to collect 30 ml of blood to reach high yields in EPC cultures.

As quick blood processing is not always available, the effect of time sampling on EPC isolation and culture procedure was checked by dividing blood samples into two sets. One set was processed within 2 hrs after extraction. The other set was left in a blood roller mixer for 24 hrs and then processed in the same way (Fig. S2). For samples processed within 2 hrs after withdrawal, 80% of EPC cultures were obtained. For those samples left for 24 hrs in a blood roller mixer, only 30% of EPC cultures were obtained (*P* < 0.05). These results indicate the critical rapid processing of blood samples when they are intended to EPC isolation.

### EPC culture conditions

**Figure 2:**
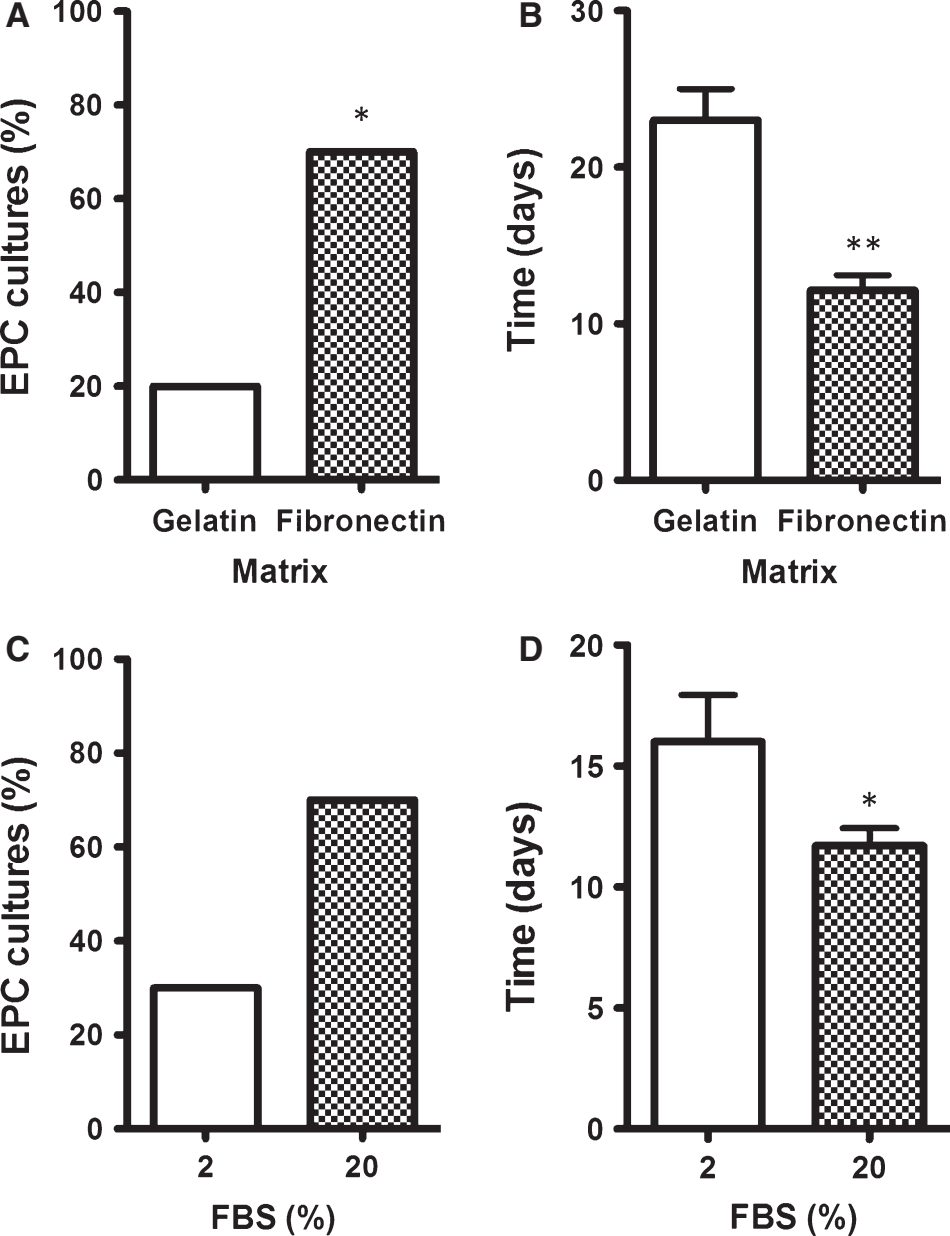
Endothelial progenitor cells' (EPC) culture conditions. Effect of culture matrix on the development of EPC cultures. Results represent (A) the obtained EPC culture percentages from mononuclear cell (MNC) seeded in plates coated with 1% gelatin or 2.5 μg/cm^2^ fibronectin (*P < 0.05 by Chi-squared test; *n* = 10) and (B) EPC appearance time (**P < 0.01 by Student's *t*-test; *n* = 10). Effect of foetal bovine serum (FBS) media concentration on the development of EPC cultures. Results represent (C) the obtained EPC cultures from MNC maintained with endothelial growing media-2 complete media supplemented with 2% or 20% FBS (*n* = 10) and (D) EPC appearance time (*P < 0.05 by Student's *t*-test; *n* = 10).

To investigate the role of the most common culture matrixes in the cell culture procedure, MNC were seeded on culture plates previously treated with either gelatin or fibronectin. The highest number of EPC cultures was obtained when fibronectin was used as coating matrix (70% *versus* 20%; *P* < 0.05; Fig. 2A). A reduction in the appearance time was also observed, from 23 days for gelatin matrix to 12 days for fibronectin matrix (*P* < 0.01; Fig. 2B). Therefore, the use of fibronectin matrix for EPC culture increases EPC culture yields as well as it reduces the appearance mean time of the first EPC colonies. The rest of the experiments were performed onto fibronectin-coated culture surfaces.

Two different concentrations of FBS, 2% and 20%, were tested as complement to endothelial media EGM-2. The higher FBS concentration higher yield in EPC cultures is obtained (30% *versus* 70%; Fig. 2C), although differences did not reach statistically significance (*P* = 0.074). Endothelial progenitor cells appearance time, however, was significantly reduced from 16 to 11 days (*P* < 0.05) for media complemented with 2% and 20% FBS, respectively (Fig. 2D).

After testing different conditions for EPC isolation and culture, the best combination of them all is the use of fibronectin as a coating matrix, a minimum volume of 30 ml of fresh blood recovered in heparin tubes and EGM-2 supplemented with 20% FBS. Blood processing within 2 hrs after extraction is also recommended. (Fig. S3 shows bright field pictures of culture evolution from MNC seeding to EPC confluent cultures).

### EPC culture appearance time and circulating levels of EPC

**Figure 3:**
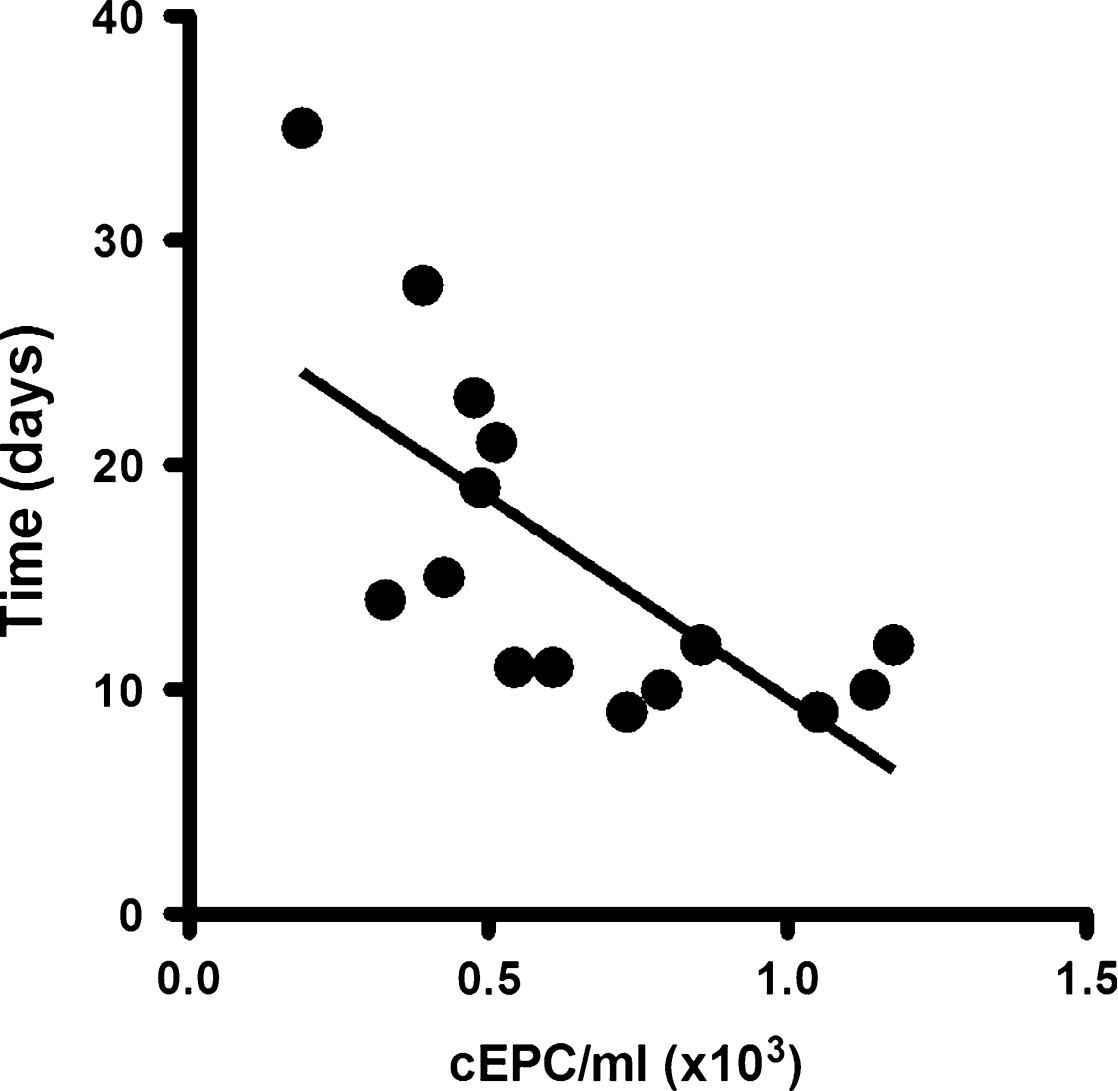
Circulating endothelial progenitor cell (EPC) levels and EPC appearance mean time relationship. Correlation between circulating EPC levels and EPC cultures appearance mean time from mononuclear cell derived from donors blood. Circulating EPC were analysed by flow cytometry as CD45^−^CD34^+^KDR^+^, as detailed in the Methods section (*R*^2^ = 0.48; *P* < 0.01; *n* = 15).

To test the relationship between cEPC and the success of its culture, blood samples from 20 healthy donors were processed in two sets: one for flow cytometry analysis for cEPC quantification, and the other one for cell culture following the above-mentioned conditions, and EPC culture yields and appearance time were recorded. With these conditions, 15 EPC cultures were obtained and the EPC appearance mean time was 13.5 days, in accordance with all the data presented so far. The cEPC levels were 647 ± 78 cEPC/ml of blood. Analysis of blood samples data that successfully gave rise to EPC cultures revealed that there was a significant, inverted correlation between cEPC levels and appearance time (*P* < 0.01; Fig. 3).

### Cultured EPC characterization

After isolation and culture procedure, obtained EPC cultures were characterized morphologically. All EPC cultures showed the characteristic endothelial cobblestone morphology. To ensure that our modified procedure gave rise to true EPC, cells were phenotypically characterized by flow cytometry and immunofluorescence microscopy.

**Figure 4:**
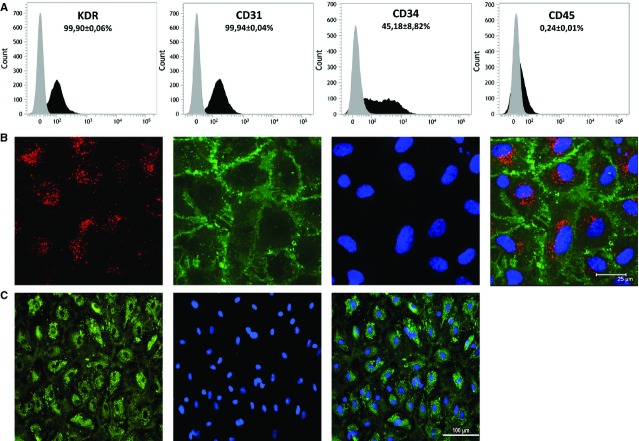
Cultured endothelial progenitor cells' (EPC) phenotypic characterization. (A) Representative images for endothelial markers kinase insert domain receptor (KDR) and CD31, the progenitor maker CD34 and the leucocyte antigen CD45 expression were assessed by flow cytometry. Grey histograms are isotype-stained cells and black histograms represent cells positively stained with KDR, CD31, CD34 and CD45. The percentage of EPC expressing each marker is presented as mean ± SEM (*n* = 10). (B) Confocal microscopy of DiI-Ac-LDL uptake, FITC-UEA-1 binding, 4′,6-diamidino-2-phenylindole (DAPI) nuclei staining and merged images for EPC are shown. EPC were incubated with 2 μg/ml of Ac-LDL for 1 hr, fixed with 4% paraformaldehyde and then incubated with 10 μg/ml FITC-Ulex-lectin. Counterstaining was achieved by 1 μg/ml DAPI staining. Scale bar represents 25 μm (original magnification ×1200). (C) Fluorescence microscopy of von Willebrand Factor expression, DAPI nuclei staining and merged images for EPC are shown. EPC were incubated for 1 hr with 1:100 dilution of FITC-vWF. Scale bar represents 100 μm (original magnification ×200). Images shown are representative of 10 EPC cultures.

Flow cytometry immunophenotyping revealed that all isolated EPC expressed endothelial markers CD31 and KDR. Panleucocyte marker expression, CD45, was absent in all of them and progenitor marker expression, CD34, was found in 45% of cultured cells (Fig. 4A). Taken together, these results suggest our procedure allows the isolation of EPC with endothelial-like phenotype.

The capacity of EPC cultures to incorporate Dil-acLDL and their ability to bind FITC- UEA-1 were analysed in different cultures by confocal microscopy (Fig. 4B). All the cultures were positive for both markers indicating an endothelial-like phenotype. Moreover, vWF expression was also evaluated by fluorescence microscopy and all the cells were uniformly positive for this marker (Fig. 4C).

Therefore, our modified culture procedure successfully achieves the isolation of EPC with endothelial cell-like phenotype.

### EPC functional parameters

To further confirm the excellence of obtained cultured EPC, the functional ability of EPC in cultures was evaluated in terms of cell growth kinetics, proliferation, adhesion and vasculogenesis. EPC functional parameters were compared with those obtained for HUVEC, the well-accepted endothelial cell model for studies *in vitro*.

**Figure 5:**
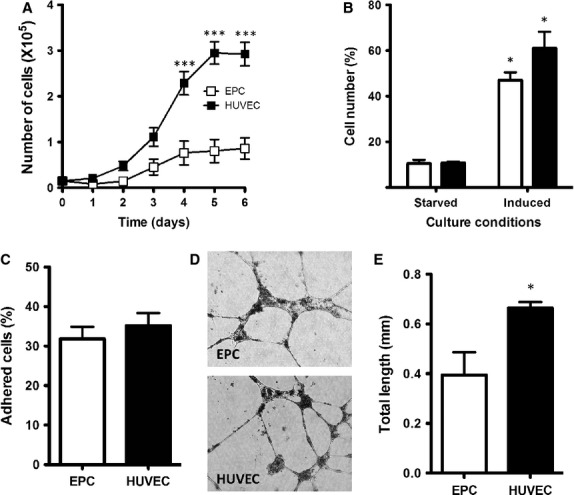
Cultured endothelial progenitor cells' (EPC) functional characterization. (A) Growth curve of EPC and human umbilical vein endothelial cell (HUVEC) cultures followed up for 6 days. 15,000 cells were seeded on day one in 24-well plates and then counted daily. Results represent the number of cells from each culture counted every day (***P < 0.001 *versus* EPC by two-way anova and Bonferroni's post- hoc test; *n* = 10). (B) Cell proliferation of EPC and HUVEC after stimulation with full endothelial growing media (EGM)-2. EPC were starved for 48 hrs and then stimulated with complete EGM-2 media for additional 18 hrs. Cells were harvested, stained with propidium iodide solution and measured by flow cytometry. Results represent the number cells in proliferation (mitosis plus interphase) for EPC (white) and HUVEC (black), expressed as a percentage of total cells (*P < 0.05 *versus* same starved cell type by Student's *t*-test; *n* = 10). (C) Cell adhesion of EPC and HUVEC. 50,000 cells were seeded on 2.5 μg/cm^2^ fibronectin-treated dishes with 4 mm^2^ grid. Results represent the percentage of attached cells 30 min. after seeding as indicated in the Methods section (*n* = 10). (D) The vasculogenesis capacity of EPC and HUVEC seeded on Matrigel is shown in the representative images of tube-like structures formed by EPC and HUVEC (original magnification ×40). 15,000 cells were seeded onto matrigel matrix diluted 1:1 with serum-free complete EGM-2 media and incubated for 8 hrs. (E) Total length analysis of tube-like structures for each sample (*P < 0.05 by Student's *t*-test; *n* = 10).

Endothelial progenitor cells and HUVEC growth curves are presented in Figure 5A. Cells were counted every day until cells reached confluence. Endothelial progenitor cells showed longer lag phase (2.4 days) compared with HUVEC (1.3 days; *P* < 0.05). Endothelial progenitor cells cultures were found to have shorter exponential growth phase. Endothelial progenitor cells plateau growth phase was reached on the 4th day after seeding at a saturation density of 5.2 × 10^5^ cells/cm^2^. By contrast, HUVEC reached plateau growth phase on the 5th day after seeding at a saturation density of 17.7 × 10^5^ cells/cm^2^ (*P* < 0.001; Fig. 5A).

Cell proliferation was studied both in starvation and after stimulation with complete EGM-2 media, after 48 hrs of starvation (Fig. 5B). In starved conditions, about 10% of cells were proliferating. When cells were stimulated with complete media, the percentage of cells actively involved in proliferation was increased four- to sixfold (*P* < 0.05 *versus* starvation). No differences in proliferation capacity between EPC and HUVEC cultures were found under starvation conditions or under induced conditions.

The cell adhesion function was evaluated by the cell ability to adhere to an extracellular matrix of fibronectin for 30 min. Results showed no differences between EPC and HUVEC (32% *versus* 35%, respectively; Fig. 5C).

Vasculogenesis, the ability to form tube-like structures, was assessed *in vitro* by seeding the cells on Matrigel matrix. All EPC and HUVEC cultures were able to organize themselves into similar tube-like structures (Fig. 5D). After 8 hrs of incubation, total length for these structures was measured. Human umbilical vein endothelial cells cultures formed larger tube-like structures (664 ± 25 μm) when compared with those formed by EPC cultures (394 ± 92 μm; *P* < 0.05; Fig. 5E).

## Discussion

This study standardizes the most controllable conditions for optimal isolation and culture of EPC from peripheral blood samples, the less invasive source for EPC available so far. Our results point to the need to control a number of parameters related to the sample collection and to the culture conditions to obtain an optimal functional performance of EPC. The conditions tested indicate (*i*) peripheral blood should be recovered in heparin tubes; (*ii*) in a minimum volume of 30 ml; (*iii*) blood samples should be processed within 2 hrs after collection; (*iv*) fibronectin is the best extracellular matrix and (*v*) culture media should be supplemented with 20% FBS.

Knowledge of EPC biology represents an advance in the understanding of vascular repair mechanisms in physiological and pathological situations. Furthermore, the fact of being able to successfully isolate and culture EPC makes us closer to test them for potential clinical applications in regenerative and anticancer therapies 12–13.

In this regard, the use of standard procedures for isolating and culturing EPC is the only way to obtain comparable results. Different culture procedures could lead to the isolation of different cell types with very different properties. A good example of this fact is the great difference in morphology, phenotype and behaviour between the so-called early and late EPC where time is the main difference condition for EPC culture and isolation (for a review, see 20) Because of the complex procedures for EPC isolation and culture, the comparison of different works is quite challenging if not sometimes impossible. There is a lack, however, of a methodical comparison of different parameters for EPC isolation and culture.

Blood recovery and handling are an extremely important step in EPC isolation as anticoagulants and sample timing handling may affect EPC integrity. In some cases, other anticoagulants have been used, although their effect on EPC isolation and culture yields, to our knowledge, has not been systematically compared. Our results suggest that correct sample handling would involve the use of heparin as anticoagulant.

An important variable when using human samples, especially to apply the procedure to patients in different circumstances, is the time spent between blood sample collection and its processing. In our study, a sample process time inferior to 2 hrs after withdrawal is mandatory to obtain the highest EPC culture yields.

Regarding culture conditions, two important parameters demonstrated an important impact on the probability to obtain an EPC culture and the mean appearance time. On the one hand, the use of fibronectin as matrix to coat cell culture superficies triplicated the success and half-reduced the appearance time, in agreement with previous reports where EPC showed a greater adherence on fibronectin matrix than collagen 1. On the other hand, the FBS, which is commonly added to cell culture media as a source of growth factors, cytokines and essential nutrients, is also critical to increase the likelihood to obtain EPC cultures and to reduce the EPC appearance mean time.

As previously reported, the volume used to isolate EPC from peripheral blood samples ranged from 50 to 100 ml 14–21. However, a minor volume of blood sample of 20 ml was required when the origin was human umbilical cord blood samples 14 probably because of a higher content in progenitor cells. Taken together, our results suppose a reduction in blood sample volumes and culture mean time than those found earlier for peripheral blood samples and a significant increase in the success rates (compared to 13% achieved in other studies 22).

Circulating EPC levels change with several physiological conditions, such as menopause 23, physical training or ageing 24. Acute disorders, such as myocardial infarction 25 or vascular trauma 26, increase the number of circulating EPC in blood samples. Conversely, an inverse correlation between EPC number and risk factors has been described in coronary artery disease 27.

The present study demonstrated an inverse correlation between the number of cEPC and EPC appearance mean time in culture, suggesting that the higher the number of circulating EPC in blood samples, the higher is the likelihood and the shorter the time to successfully obtain EPC cultures out of them. This information is of great importance if our proposed procedure is intended for EPC isolation, culture and cell therapy. In this case, previous EPC mobilization would be strongly recommended to ensure high yields and time reduction in EPC cultures, particularly in those pathologies where EPC numbers have been reported to be reduced 27.

But not only is the yield of the method important but the functional characteristics of the obtained EPC. The morphological, phenotypical and functional characteristics of cultured EPC were comparable to endothelial cells from other sources, such as HUVEC. Cultured EPC showed a cobblestone-like morphology was positive for LDL uptake and Ulex-lectin binding, expressed progenitor antigen CD34 as well as endothelial antigens CD31, KDR, vWF while lack the expression of hematopoietic marker CD45. Our procedure give rise to EPC with similar traits to the so-called late EPC, endothelial colony forming cells or endothelial outgrowth cells 11–21.

Endothelial progenitor cells obtained under the best conditions exhibited functional parameters comparable to those obtained for HUVEC in terms of growth curve, cell proliferation and adhesion and vasculogenesis. In spite of cultured EPC showed reduced growth (which can be attributed to the longer lag phase) and vasculogenic capabilities than HUVEC, no differences were found in proliferative and adhesive capabilities between these two cell types. Therefore, the culture conditions provided are not only an adequate tool to study EPC but also a source of endothelial cells from adults.

The agreement on EPC definition (CD34^+^KDR^+^ phenotype) represents the best compromise in terms of detection accuracy, biological meaning and clinical usefulness 28, as well as the development of standardized procedures for EPC isolation and culture opens the use of EPC for clinical application.

In summary, we presented a standard protocol for EPC isolation and culture from human peripheral blood. Additional *in vivo* testing of the isolated EPC following our procedure must be performed to fully characterize the possible potential of this cell population for cell therapy use and regenerative medicine.

## Supporting Information

**Figure S1** Phenotypic characterization of cells isolated from citrate tube collected blood. Bright field images of cell cultures (A) ×40 and (B) ×200 magnification. Fluorescence microscopy of DiI-Ac-LDL uptake (C), FITC-UEA-1 binding (D), DAPI nuclei staining (E) and merged images (F) are shown. Cells were incubated with 2 μg/ml of Ac-LDL for 1 hr, fixed with 4% paraformaldehyde and then incubated with 10 μg/ml FITC-Ulex-lectin. Counterstaining was achieved by 1 μg/ml DAPI staining. Scale bar represents 100 μm (original magnification for fluorescence microscopy images: ×200). **Figure S2** Influence of processing time on the success of EPC cultures. Blood samples were divided into two sets. One was processed within 2 hrs and the other 24 hrs after withdrawal. The success of EPC culture was expressed as the percentage of EPC cultures obtained (*P < 0.05 by Chi-squared test; *n* = 10). **Figure S3** MNC culture and EPC isolation. Representative images of EPC cultures. (A) MNC after seeding. After 24 hrs of incubation, non-adhered cells were removed and attached cells (B) were further cultivated. On day 15 of culture, first EPC colonies appeared (C). EPC colonies were cultured for 7 days or until they reached confluence (D). All the pictures above shown were taken at ×100 magnification. **Table S1** Characteristics of healthy individuals.
