# Supplementary material for: An affordable method to obtain cultured endothelial cells from peripheral blood
Source: J Cell Mol Med. 2013 Oct 1;17(11):1475–83. doi: 10.1111/jcmm.12133 (PMC4117560; doi:10.1111/jcmm.12133)

Supplemental Table 1: Characteristics of healthy subjects

Variable	Subjects (n= 60)
Male (%)	78.3
Age (years)	55.0±1.4
Weight (Kg)	75.6±1.2
BMI (Kg/m^2)	25.6±0.3
Smokers (%)	22.1
Total cholesterol (g/L)	189.0±2.3
HDL cholesterol (g/L)	53.9±1.2
LDL cholesterol (g/L)	118.7±2.1
Triglycerides (g/L)	81.9±4.4
SBP (mmHg)	119.4±1.4
DBP (mmHg)	74.0±1.1

BMI indicates body mass index; HDL, high density lipoprotein; LDL, low density lipoprotein; SBP, systolic blood pressure; DBP, diastolic blood pressure.

Supplemental Figure 1

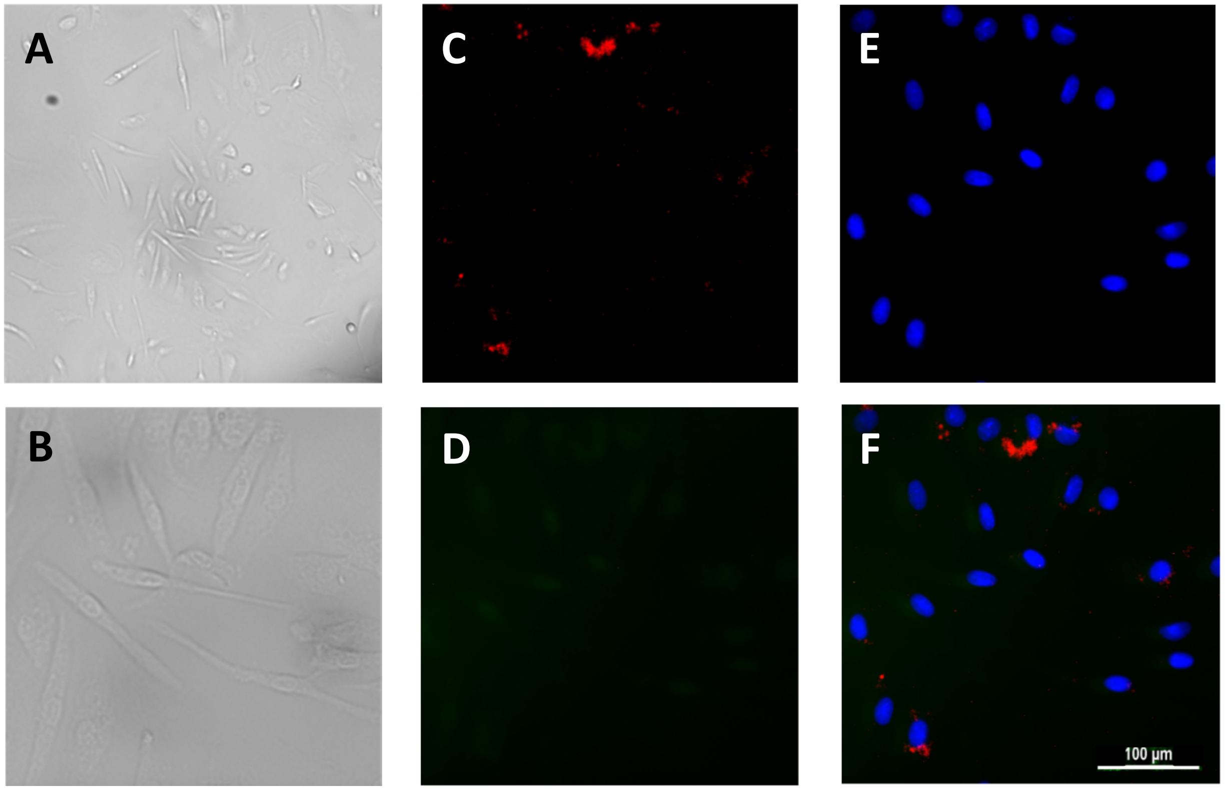

Supplemental Figure 2

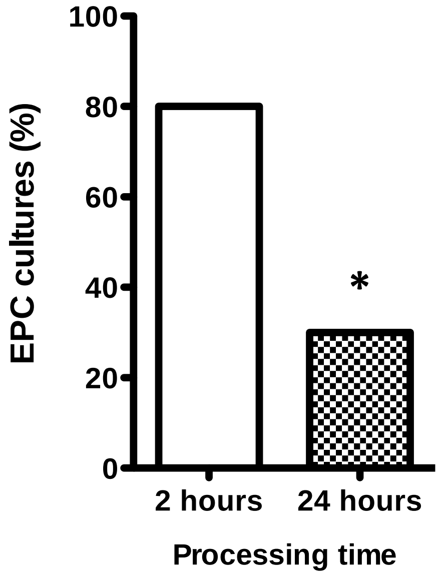

Supplemental Figure 3

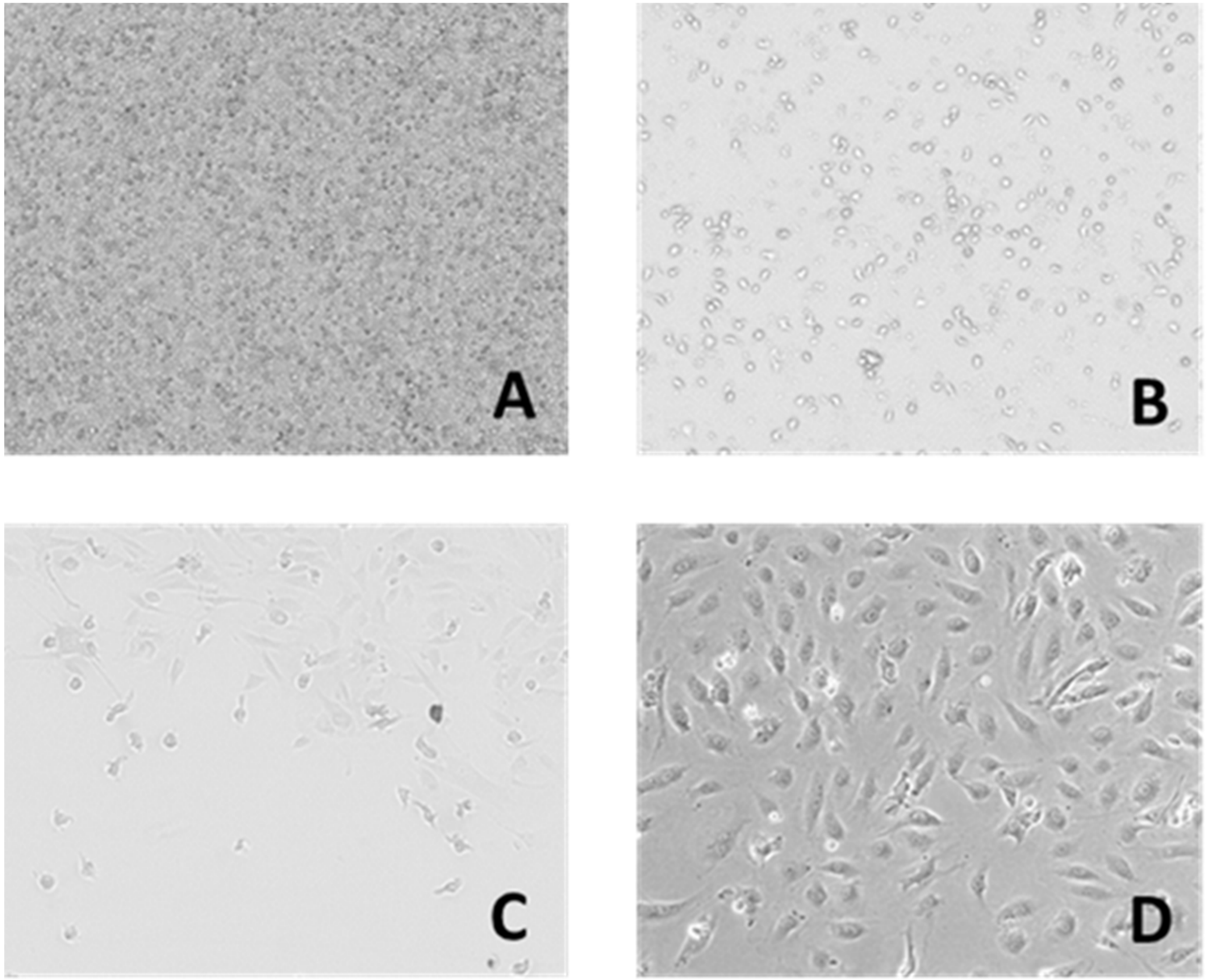

Supplement: Supplementary file 1 — Figure S1 Phenotypic characterization of cells isolated from citrate tube collected blood. Bright field images of cell cultures (A) ×40 and (B) ×200 magnification. Fluorescence microscopy of DiI-Ac-LDL uptake (C), FITC-UEA-1 binding (D), DAPI nuclei staining (E) and merged images (F) are shown. Cells were incubated with 2 μg/ml of Ac-LDL for 1 hr, fixed with 4% paraformaldehyde and then incubated with 10 μg/ml FITC-Ulex-lectin. Counterstaining was achieved by 1 μg/ml DAPI staining. Scale bar represents 100 μm (original magnification for fluorescence microscopy images: ×200). Figure S2 Influence of processing time on the success of EPC cultures. Blood samples were divided into two sets. One was processed within 2 hrs and the other 24 hrs after withdrawal. The success of EPC culture was expressed as the percentage of EPC cultures obtained (*P < 0.05 by Chi-squared test; n = 10). Figure S3 MNC culture and EPC isolation. Representative images of EPC cultures. (A) MNC after seeding. After 24 hrs of incubation, non-adhered cells were removed and attached cells (B) were further cultivated. On day 15 of culture, first EPC colonies appeared (C). EPC colonies were cultured for 7 days or until they reached confluence (D). All the pictures above shown were taken at ×100 magnification. Table S1 Characteristics of healthy individuals. [file jcmm0017-1475-sd1.pdf]
